# Genetic and Epigenetic Somatic Alterations in Head and Neck Squamous Cell Carcinomas Are Globally Coordinated but Not Locally Targeted

**DOI:** 10.1371/journal.pone.0009651

**Published:** 2010-03-11

**Authors:** Graham M. Poage, Brock C. Christensen, E. Andres Houseman, Michael D. McClean, John K. Wiencke, Marshall R. Posner, John R. Clark, Heather H. Nelson, Carmen J. Marsit, Karl T. Kelsey

**Affiliations:** 1 Departments of Molecular Pharmacology and Physiology, Brown University, Providence, Rhode Island, United States of America; 2 Departments of Pathology and Laboratory Medicine, Brown University, Providence, Rhode Island, United States of America; 3 Department of Community Health, Center for Environmental Health and Technology, Brown University, Providence, Rhode Island, United States of America; 4 Department of Biostatistics, Harvard School of Public Health, Boston, Massachusetts, United States of America; 5 Department of Environmental Health, Boston University, Boston, Massachusetts, United States of America; 6 Department of Neurological Surgery, University of California San Francisco, San Francisco, California, United States of America; 7 Department of Medical Oncology/Solid Tumor Oncology, Dana-Farber Cancer Institute, Boston, Massachusetts, United States of America; 8 Department of Medicine Hematology/Oncology, Massachusetts General Hospital, Boston, Massachusetts, United States of America; 9 Division of Epidemiology and Community Health, Masonic Cancer Center, University of Minnesota, Minneapolis, Minnesota, United States of America; Deutsches Krebsforschungszentrum, Germany

## Abstract

**Background:**

Solid tumors, including head and neck squamous cell carcinomas (HNSCC), arise as a result of genetic and epigenetic alterations in a sustained stress environment. Little work has been done that simultaneously examines the spectrum of both types of changes in human tumors on a genome-wide scale and results so far have been limited and mixed. Since it has been hypothesized that epigenetic alterations may act by providing the second carcinogenic hit in gene silencing, we sought to identify genome-wide DNA copy number alterations and CpG dinucleotide methylation events and examine the global/local relationships between these types of alterations in HNSCC.

**Methodology/Principal Findings:**

We have extended a prior analysis of 1,413 cancer-associated loci for epigenetic changes in HNSCC by integrating DNA copy number alterations, measured at 500,000 polymorphic loci, in a case series of 19 primary HNSCC tumors. We have previously demonstrated that local copy number does not bias methylation measurements in this array platform. Importantly, we found that the global pattern of copy number alterations in these tumors was significantly associated with tumor methylation profiles (*p*<0.002). However at the local level, gene promoter regions did not exhibit a correlation between copy number and methylation (lowest *q* = 0.3), and the spectrum of genes affected by each type of alteration was unique.

**Conclusion/Significance:**

This work, using a novel and robust statistical approach demonstrates that, although a “second hit” mechanism is not likely the predominant mode of action for epigenetic dysregulation in cancer, the patterns of methylation events are associated with the patterns of allele loss. Our work further highlights the utility of integrative genomics approaches in exploring the driving somatic alterations in solid tumors.

## Introduction

Head and neck squamous cell carcinoma (HNSCC) is the eighth most commonly diagnosed malignancy in males, responsible for over an estimated 11,000 deaths each year in the United States [1]. The genetic alterations common to HNSCCs have been characterized using both cytogenetic and molecular approaches: Importantly, the presence of genetic imbalances, specifically loss in chromosomal regions 3p, 8p, 9p, 15p, 18q, 22q and gains in 1q, 3q, 8q, 11q, 14q, 16q, 20q have been shown to be significantly associated with poor patient survival [2], [3], [4], [5], [6]. Epigenetic alterations commonly observed in this disease include promoter hypermethylation, resulting in gene silencing, of *CDKN2A*, *CDH1*, *DAPK1*, *RASSF1*, and *MGMT*, which have been shown to be associated with patient outcome [7], [8], [9]. Evidence has emerged that *CDKN2A* [10], [11], *RASSF1* [12], and other genes are regulated both by hypermethylation and allele loss in many solid tumor types [13], [14], [15] leading to the hypothesis that, in addition to classical Knudson inactivation of tumor suppressors through mutation [16], first and second hits commonly occur in the form of promoter methylation and loss of heterozygosity (LOH) even in the absence of mutation.

The combination of genetic and epigenetic alterations is fundamental in the genesis of neoplasia, resulting in the inappropriate activity level of cell signaling pathways that regulate key processes such as cellular growth and differentiation, DNA fidelity, apoptosis, and metabolic stability. Thus, a more complete study of carcinogenesis would include simultaneous evaluation of multiple types of alterations in common tumors. Investigation of various cancers using genome level technologies, such as high-resolution single nucleotide polymorphism (SNP) microarrays to measure somatically arising allelic imbalance, have shown that these genetic alterations profiles are remarkably diverse [3], [17], [18]. Further, recent large scale array-based studies of epigenetic events have yielded similar insight into the pattern of gene silencing in cancers, in that alterations to promoter methylation status of genes occurs in a highly variable pattern even amongst tumors arising from the same tissue or cell type [19], [20], [21]. Results from studies employing these methods have been effective in gaining insight into the basis of hereditary disease [22] and in identifying novel candidate cancer genes [23].

Our technological capability to assess both genetic and epigenetic genome-wide alterations has improved. Thus, it is now critically important to begin integrated analyses that will allow us to define the relationship between epigenetic alterations (represented by changes in DNA methylation) and genetic changes (represented by alterations in copy number) that comprise the etiologic keystones of malignant disease. The need for combined high-resolution profiling of DNA copy number and methylation profiling is becoming recognized, particularly as pharmacologic targeting of the epigenome has gained momentum, and methods to simultaneously investigate both types of alterations are emerging [24], [25], [26]. In addition, recent investigations in gliomas [21] and cancer cell lines [27], [28] using combinatorial high-throughput methods have elucidated individual genes that are differentially regulated through these mechanisms; however, the global relationships between epigenetic and copy number profiles in human tumors remain poorly characterized.

We hypothesized that epigenetic and genetic alterations in HNSCC are clonally selected in a fashion that is not independent. To investigate this, we have integrated these genomic-level data in an analysis of 19 primary HNSCCs.

## Results

### DNA Copy Number and Methylation Measurement

**Figure 1:**
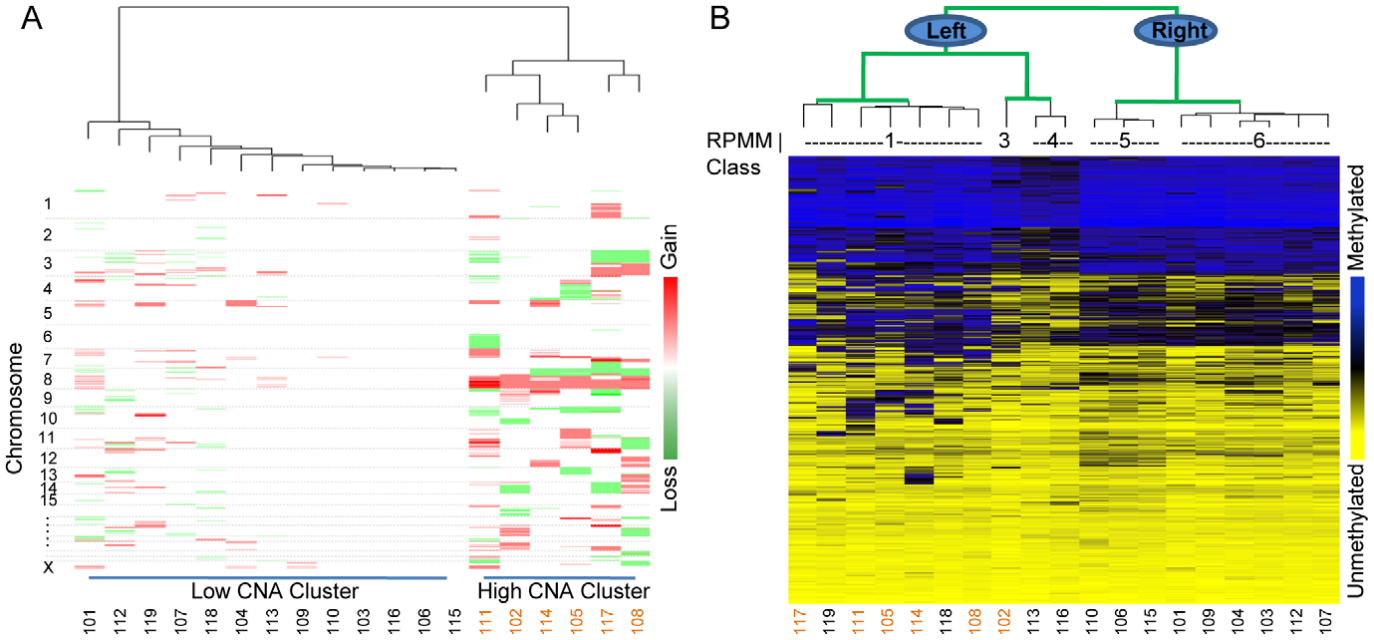
Hierarchical clustering heatmaps for 19 HNSCC tumors. A) DNA copy number states are arranged by chromosome for 500,000 SNP loci. Copy number is red for amplified regions up to 4+ copies, white for 2 normal copies, and green for allele loss to 0 copies. Tumors are ordered by unsupervised hierarchical clustering and are dichotomized into low/high CNA clusters. B) Methylation loci (more methylated = blue, less methylated = yellow) are grouped by Euclidean distance and tumors samples are ordered first by RPMM class structure (green branches) then by simple hierarchical clustering (black branches). Tumor IDs are provided below each plot and “high CNA” samples are colored orange for reference.

Somatic DNA copy number analysis was performed on a representative case-series of 19 malignant HNSCCs (Table 1) with high-density Affymetrix 500 k SNP mapping arrays, using matched blood DNA as referents. For the purpose of exploration, copy number data were subjected to unsupervised hierarchical clustering (Ward's method with Hamming distance) and coordinately arranged by chromosome (Figure 1A). Consistent with the considerable literature addressing the cytogenetics of HNSCC [29], [30], frequent gross structural abnormalities of chromosomes 8q or 3q are observed in 6 (32%) of the cases, appearing as amplifications (red) or allele losses (green), while smaller-scale aberrations are identifiable in most samples.

**Table 1:** Clinicopathologic characteristics of HNSCC study participants.

	All Cases (n = 68)	Cases For Methylation and Copy Number Analysis (n = 19)
Gender, n (%)		
Female	14 (21)	4 (21)
Male	54 (79)	15 (79)
Age at Diagnosis		
Range	25–85	25–85
Mean (SD)	58 (11.4)	59 (15.1)
HPV16 Status, n (%)		
Positive	10 (15)	3 (16)
Negative	56 (82)	16 (84)
Tumor Site, n (%)		
Oral	35 (52)	15 (79)
Pharynx	26 (38)	2 (10.5)
Larynx	7 (10)	2 (10.5)
Clinical Stage, n (%)		
I	5 (7)	2 (10.5)
II	9 (13)	2 (10.5)
III	9 (13)	5 (26)
IV	26 (38)	9 (47)
Unknown	19 (28)	1 (5)

Previously, we reported the methylation status of 1505 CpG loci using the Illumina GoldenGate platform in 68 HNSCC tumors (including the 19 samples with copy number data) [20]. Employing an unsupervised method for clustering methylation data using a mixture of beta distributions, termed recursively partitioned mixture modeling (RPMM) [31], we showed that normal epithelium is distinguished from tumor in the classifications (epigenetic signatures) that result. When restricting to tumor-only modeling, six methylation profile classes were defined, and class membership was significantly associated with tumor stage (*p*<0.01), patient age (*p*<0.01), and marginally associated with tumor site (*p*<0.10) and Human Papillomavirus (HPV) status (*p*<0.10) by permutation tests [20]. Tumor membership in Class 5 carried an increased risk of high stage disease while Classes 6 and 2 were associated with a protective effect against advanced stage. Patients in Class 4 had a higher prevalence of HPV16 positivity and Class 3 had the highest proportion of laryngeal tumors. These associations lend biological significance to the six epigenetic profiles that were identified. Subsequent analyses presented in this report utilize these previously published methylation classifications. Loci profiled for methylation (hypermethylated = blue, hypomethylated = yellow) for the 19 tumors with copy number measurements were visualized by clustering (Figure 1B) and are ordered by their corresponding placement within a dendrogram obtained by RPMM grouping, with the terminal nodes filled by Ward's method of hierarchical clustering. RPMM classes are indicated beneath the dendrogram.

### Local Molecular Alterations Are Not Correlated

**Figure 2:**
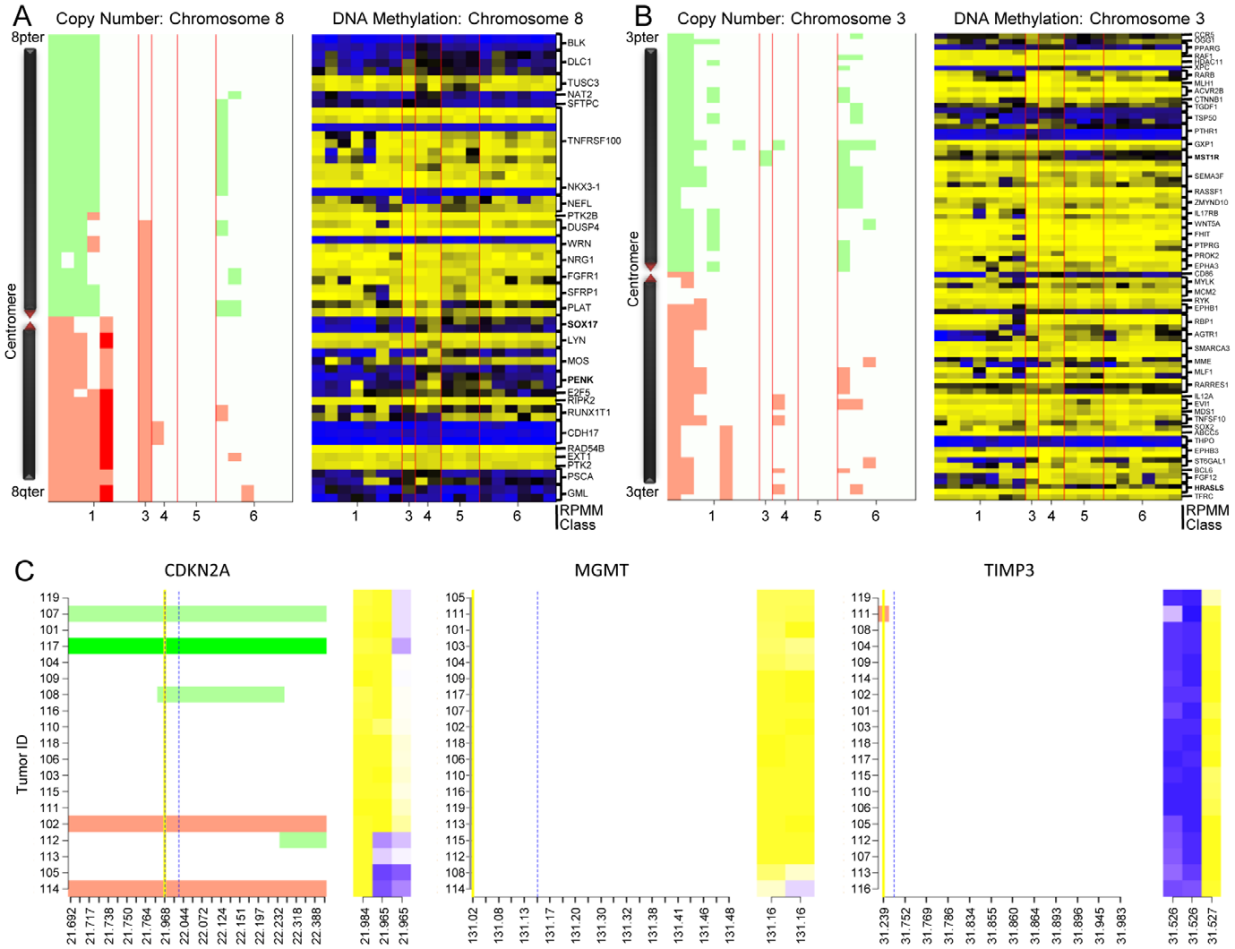
Integration of copy number and methylation values for 19 HNSCCs. Tumor profiles in combined color image plots for A) chromosome 8 and B) chromosome 3 are grouped by RPMM-modeled methylation classes. Methylation values (more methylated = blue, less methylated = yellow) are located to the right, while copy number (red = gain, green = loss) is displayed on the left within each panel. SNP loci are matched to the nearest CpG locus and these matched loci (common to both subpanels) are oriented coordinately along the chromosomes. For legibility, the loci are grouped by gene. Note: Chromosome diagrams are not to scale, but centromeric regions are correctly located between genes. C) Gene-focused copy number and methylation data. The x-axis displays the genomic coordinate (x10^-6^) for each locus. Vertical yellow lines mark the TSS and dashed blue lines denote locations of the mapped CpG loci, whose values are displayed on the right portions of the plot for each gene. Methylation average β is bright blue for fully methylated and bright yellow for unmethylated. Copy number is displayed on the left plot and SNP loci marked red represent amplified alleles and green for deleted allele calls.

To compare methylation levels and copy number alteration (CNA) in greater detail, we generated integrated color image plots of methylation and copy number profiles for individual genomic regions around specific loci (Figure 2C) and entire chromosomes of interest (Figure 2A/B and Figure S1), where samples were grouped by RPMM methylation class membership. These plots illustrate the local relationships between DNA methylation and CNA. Specifically, certain loci (e.g. *SOX17*, chromosome 8) in tumors with allelic amplification exhibit hypermethylation in nearby CpGs compared to those tumors without allelic imbalance in that region. At the same time, methylation values were stable for most loci across all the samples despite local regions of gain or loss, demonstrating that, as we have previously reported [32], the relationship between methylation profile and CNA is neither an artifact of the analysis nor allelic bias in the samples.

Initial analyses sought to take advantage of “two hit” gene inactivation, as proposed by Knudson [16], in order to identify potential novel loci as candidates that are causal in this disease. We scanned the genome for locations where there was a systematic relationship between copy number and methylation, such as previously reported sites of hypermethylation and LOH. Calculating the Pearson correlations for all overlapping loci, we encountered only eight loci (*q*<0.05) that demonstrated a significant correlation between these disparate mechanisms of gene silencing. This apparent independence was true both at individual loci and when averaged over multiple CpGs upstream of the transcriptional start site (TSS) (Table 2). For example, hypermethylation and LOH occurred infrequently within the *CDKN2A* gene and only at one CpG, while more often DNA methylation occurred in the absence of aberrant copy number states or vice-versa (Figure 2C). Importantly, many other loci (such as those within *MGMT*) had little variation in either form of molecular alteration.

**Table 2:** Methylation and CNA-correlated regions.

	Estimate*	*p*-value	*q*-value
Locus			
GRB10_P260_F	0.96	<1.0E-04	0.01
GRB10_E85_R	0.94	<1.0E-04	0.01
IHH_P529_F	0.99	<1.0E-04	0.01
HOXA11_P92_R	0.94	1.0E-04	0.02
DDR2_E331_F	−0.91	2.0E-04	0.03
TERT_E20_F	0.85	2.0E-04	0.03
FZD9_P15_R	0.85	4.0E-04	0.03
WNT1_P79_R	0.86	4.0E-04	0.03
PI3_P274_R	−0.82	5.0E-04	0.05
IHH_P246_R	0.82	7.0E-04	0.05
Gene Promoter			
HOXA11	0.94	1.0E-04	0.04
NID1	−0.75	1.4E-03	0.12
GRB10	0.73	1.8E-03	0.12
TIMP3	−0.75	2.0E-03	0.12
AFP	−0.71	2.2E-03	0.12

*Point estimates represent Pearson product-moment correlation coefficients.

**Figure 3:**
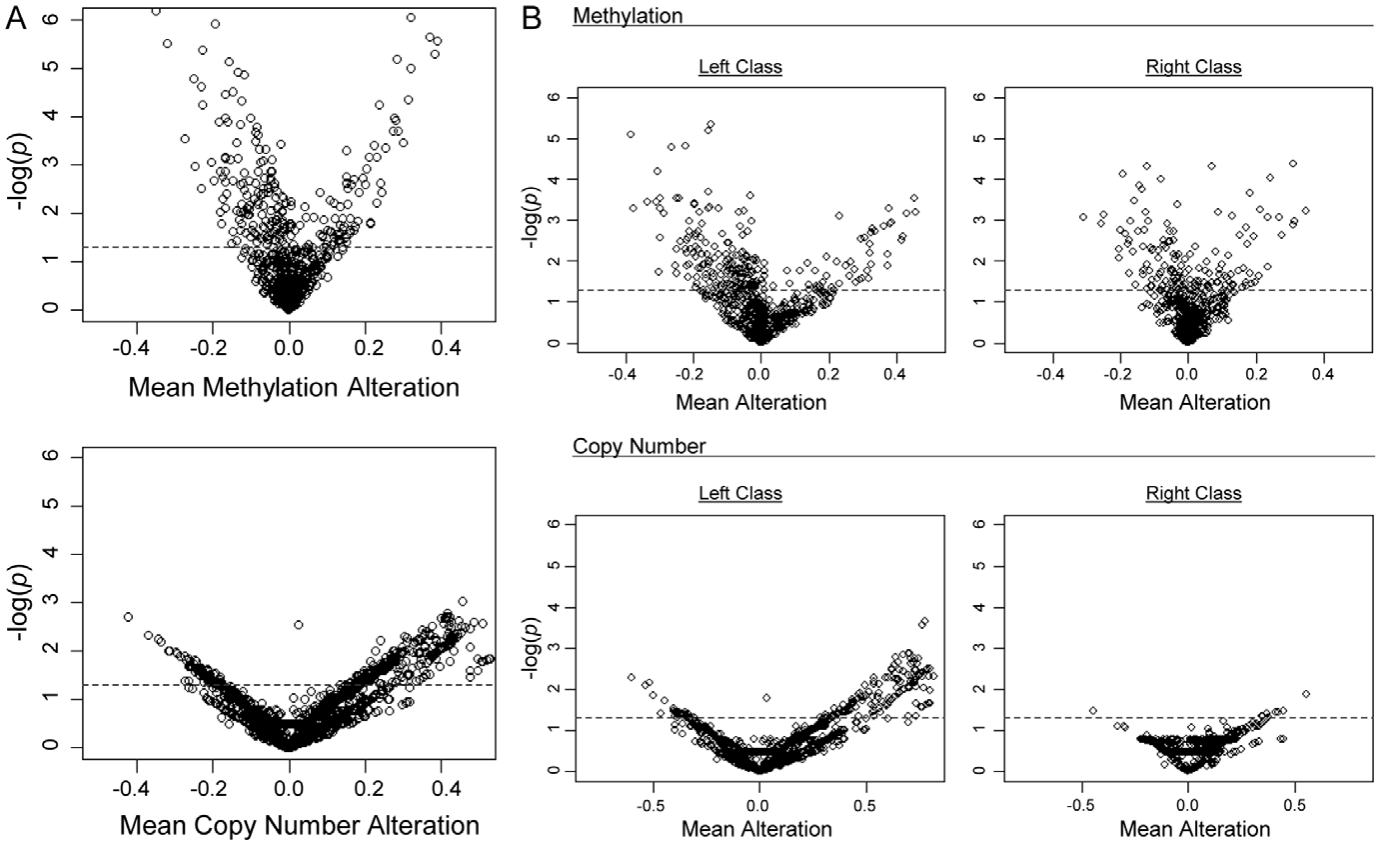
Log significance plots for mean alteration difference in array genes. Gene regions were compared to their expected value (normal tissue betas for methylation and copy number = 2 for CNA) and *t*-tests were performed. Negative log-transformed *p*-values (generated by tumor/normal *t*-tests) are shown on the y-axes and the indicated mean alterations are displayed in the x-dimension. The space above the dotted line represents a significance level of *p*<0.05. A) Promoter-associated methylation alterations (663 genes) and copy number-altered genes (n = 15,790) are shown and B) separated by overall methylation class structure (Left Class n = 10, Right Class n = 9) as defined by grouping the methylation classes based on the original RPMM dendrogram splitting in [20].

We next investigated each form of alteration individually, and estimated the deviations from their expected normal values to determine the significance of CNA and DNA methylation alterations in HNSCC. Volcano plots of gene-specific mean methylation alteration and mean copy number alteration versus *p-*value revealed distinctions in promoter-associated alterations between molecular processes, evident through an increased tendency for significant loss of methylation versus a tendency for significant increase in copy number (Figure 3A and Table S1). To further explore allele losses in the context of the overall process of methylation at the specific loci on the array, these data were stratified by their RPMM methylation classification. Specifically, “left class” included tumors in RPMM Methylation Classes 1–4, while “right class” included Methylation Classes 5–6. These groupings represent the two main epigenetically distinct methylation class subsets and are defined as the initial left and right splits of the RPMM clustering dendrogram, shown in Figure 1B, as adapted from [20]. Interestingly, the pattern of allelic copy number and methylation alterations differed considerably between right and left classes (Figure 3B), with significantly decreased levels of methylation alterations and significantly increased CNA occurring primarily in the left class. This provides evidence that different global processes are at work between the groups of methylation classes and that this distinction is replicated in the copy number data.

### CNA and Methylation Profiles Are Not Independent

**Figure 4:**
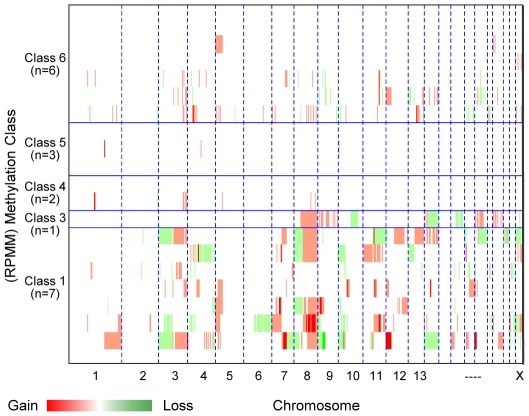
Copy number plots by RPMM class. Copy number data for 19 tumors are coordinately arranged by chromosome and grouped together according to RPMM-modeled methylation classes. The total number of methylation classes is reduced from the original six [20] to five in this study because membership in Class 2 methylation profile is not represented in the subset of 19 tumors investigated.

In order to more fully explore the evidence of a global epigenetic effect on copy number, we plotted genome-wide allele copy number changes in tumors stratified by RPMM methylation class membership (Figure 4). The extent of copy number alterations varied significantly by methylation class (permutation test *p*<0.002), with tumors in Methylation Classes 1 and 3 showing substantial, large-scale copy number alteration relative to other tumors. This clearly shows that copy number and methylation alterations do not occur independently. If there were no association between genetic and epigenetic alterations, one would observe an even distribution of aberrant copy number states across methylation classes. To investigate the notion that clinical variables previously shown to be associated with the methylation classes may also be the reason for similar copy number data clustering, tests for association between the degree of CNA and the clinical covariates age, site, stage, and HPV16 status were performed. Importantly, these tests were not statistically significant, further indicating that a relationship between theses global processes of regulation exists rather than purely a manifestation of clinical parameters in both datasets.

### Global Methylation in High/Low CNA Tumors

Since it has long been hypothesized that genomic instability is related to decreased levels of global DNA methylation, we measured LINE-1 methylation, as a surrogate marker of global methylation, in tumors with available DNA (n = 11). The tumors that hierarchically clustered in the high CNA group (see Figure 1A) had generally lower LINE-1 methylation than tumors with low levels of allelic imbalance (mean differential methylation: −13.2%, 95% CI: (−33.6%–7.2%)).

## Discussion

We recently constructed epigenetic profiles of HNSCC, reporting that DNA methylation events are common and associated with etiologically important exposures [20]. Aberrant DNA methylation events have been hypothesized to accumulate initially in a stochastic fashion and, through positive selection, result in clones that have a growth advantage that leads to the genesis of a rapidly-dividing tumor. Here we expand upon these data and include an analysis of chromosomal integrity in these same tumors. Using genomic-level measurements, we observed a highly significant association between copy number and DNA methylation profiles, definitively showing that these modes of gene regulation are linked in HNSCC. These observations supplement recent evidence from Sadikovic *et al.* that copy number alterations are generally correlated with both methylation and gene expression levels in osteosarcomas [33]. At the same time, while specific targeting of genes through both mechanisms occurs in a deterministic manner within subgroups of patients, when we tested for regionally matching local (gene level) epigenetic and copy number events we only observed that global, rather than local, alterations were correlated. This indicates that coordinated two-hit gene inactivation (LOH followed by epigenetic silencing) is not the dominant character of somatic alteration over the genome. As the GoldenGate methylation array investigates nearly 800 cancer-involving genes and is enriched for tumor suppressor-associated loci, we were uniquely positioned to investigate just this question. Recent evidence supports our conclusion, as gene regulation by CNA and DNA methylation measured at 691 loci in meningiomas appears to be somewhat mutually exclusive [34]. In addition, our combined analysis of the promoter regions of previously reported genes with allele loss or hypermethylation demonstrates that this situation is rare (see Table S2), however a much larger investigation with higher resolution is needed to determine if these alterations occur systematically.

One possible explanation for the association between global profiles of DNA methylation and copy number is that amplification or loss of genetic material may result in a bias of measured methylation for CpGs within that region, potentially contributing to the inferred methylation profile (e.g. in our RPMM approach). Indeed, previous microarray-based methods to determine methylation status have been hindered by copy number changes that bias the measured relative methylation values at CpG loci [35]. However, our recent work utilizing bead-arrays has shown that CNA produces little bias in absolute methylation data generated on the GoldenGate methylation panel, except in the case of homozygous deletion [32]. We and others have previously demonstrated the validity of Illumina GoldenGate methylation array results with other high- and low-throughput technologies [36], [37].

Integrative analysis revealed that several tumors with similar methylation profiles had large regions of chromosomal abnormality, particularly in chromosomes 8 and 3, consistent with the possible formation of isochromosomes i(8q) and i(3q) in aneuploid cells. These cytogenetic abnormalities commonly appear in HNSCC, possibly a result of chromosomal missegregation events during mitosis [29]. We also observed that Methylation Class 3 tumor data reflect gross allelic amplification of 8q, which extends through the centromere and partially into 8p, possibly indicating a distinct mechanism of formation for this anomaly. Among tumors with an amplified 8q arm, several methylated CpG loci were observed in this region relative to tumors without this gross chromosomal alteration. Two mechanisms can be posited to explain this result. Firstly, epigenetic dysregulation may occur early in the genesis of these head and neck tumors and aberrant methylation marks are faithfully replicated despite the amplification event, which is consistent with previous reports implicating epigenetic modification as an early event in the progression of this disease [reviewed in 38]. In fact, there is evidence that aberrant methylation in certain chromosomal regions, especially located near centromeres, predisposes the surrounding area to genetic alteration, including fragile breakpoint sites [39], [40]. On the other hand, it is possible that this differential methylation occurs following the chromosomal aberration, possibly in response to the genetic event and selective pressures. However, we are unable to distinguish between these possibilities in our data, highlighting the need for mechanistic studies.

Gain of 8q has been reported as a relatively common event in HNSCC, particularly at 8q24 [41], which houses the *MYC* oncogene, and 8q22, thought to be targeting *LRP12* [42]. Similarly, in one-third of our cases we observed amplification of this entire arm, while putative tumor suppressor genes, such as *SOX17* and *PENK*, within this amplified chromosomal arm are methylated. These findings are suggestive of a context wherein genetic modification (possibly a result of genomic instability) is responsible for perpetuating inappropriate oncogene expression with concomitant epigenetic silencing of local tumor suppressors (Figure 2A).

Molecular alterations in chromosome 3 have been previously reported as the most prevalent and potentially most important in HNSCC [43]. Consistent with these findings, we observed extensive copy number and methylation alterations in this chromosome. For example, the gastric cancer-associated tumor-suppressor *HRASLS* in the amplified q-arms were more highly methylated than those tumors which possessed normal 3q. In addition, the proto-oncogenic *MST1R* loci, associated with poor prognosis through potentiation of cell scattering and invasion in breast cancer [44], were unmethylated in most tumors irrespective of chromosome 3p loss. However, we observed a number of genes that did not follow the expected directions of methylation within copy number variable loci, indicating that they may be hitchhikers or simply regulated by other genetic or epigenetic means. Overall, these structural modifications in chromosomes 3 and 8 are consistent with the literature and are thought to develop early during the genesis of disease [30], [45].

Although 13 of the tumors examined (Methylation Classes 1, 3, and 6, Fig. 4) demonstrated a preponderance of CNA, we observed a notable lack of CNA among the remaining tumors. We hypothesize that this may be due to these samples having higher levels of aberrant epigenetic or non-copy number altering genetic events such as mutation or chromosomal rearrangements. It is also possible that clinical stage could account for the observed levels of abnormal copy number, as this has been reported in other cancers [46]. Other possible confounders include HPV16 status and tumor site, although our data do not indicate associations between any of these covariates and CNA. Larger future studies are required to investigate the nature of these notable differences with statistical rigor.

There was also an apparent relationship between global hypomethylation, represented by the extent of LINE-1 DNA sequence methylation, and the increased levels of allelic imbalance among HNSCC cases. This finding is consistent with the literature [47], [48] and with the hypothesis that global hypomethylation of transposable elements culminates in genomic instability. While it is apparent that the various modes of alteration are related, the timing of these events is less clear. Our data underscore the need for additional investigations into the chronology of multifaceted somatic alterations leading to the onset and progression of this deadly disease.

In our analysis to define the local relationships between copy number and methylation, we observed only one gene (where promoter-associated CpG alterations were averaged), *HOXA11*, with a marginally significant correlation between methylation and copy number alteration, although a number of individual CpG loci reached significance, including sites within potentially oncogenic *GRB10, IHH, and HOXA11*. While the strong positive correlation at these sites could indicate selection pressure for dual mechanism inactivation was occurring to promote neoplasm formation, there was little evidence of this pressure acting over the entire set of measured genes. Our finding that different genes are preferentially targeted through different mechanisms in HNSCC could reflect dramatic differences in the timing of these events (e.g. one type of somatic changes predominating early in clonal evolution with the other becoming dominant later in clonal evolution). Alternatively, it is possible that other simultaneous genetic events obviated the need for epigenetic modifications (e.g. copy number-activating mutations common in other cancers [49]) or that sequence context (e.g. proximity to fragile sites or the CpG content of promoter regions) may interact with carcinogen exposure to select the order and the type (epigenetic or genetic) of alteration that inactivates genes. At the same, our stratified analysis of the two main biological methylation subgroups revealed that the events leading to abnormal copy number and CpG methylation are fundamentally different in each group, suggestive of an overall collateral relationship.

In sum, epigenetic profiles in HNSCC are significantly associated with the extent of CNA, but this global relationship is not widely reflected at the local level. Furthermore, the molecular targets of each are dissimilar. The precise mechanisms responsible for gene inactivation are obscure but in the framework of carcinogenic progression within Knudson's two-hit model, our data indicate that local, coordinate DNA methylation and copy number alteration do not dominate the profile of changes in primary HNSCC.

## Materials and Methods

*Study Population/Ethics*. The study group were members of a case-control population presenting at Boston-area hospitals from 2000–2004, as previously described [50]. In short, samples from incident cases of HNSCC were microscopically examined and histologically confirmed to have >75% tumor content by the study pathologist. This study was conducted according to the principles expressed in the Declaration of Helsinki. Selected patients were enrolled upon providing written, informed consent. All protocols and documentation were approved the Brown University institutional review board administered through the Research Protections Office (Protocol #0707992334). Clinical information was collected and HPV16 status was assessed using short fragment PCR to amplify a region of the *L1* gene of HPV16, according to previously published methods [51].Tumor specimens from all head and neck sites (excluding glandular, nodal, and nasopharyngeal carcinomas) selected for CpG methylation analysis included 26 fresh-frozen samples and 42 formalin-fixed paraffin-embedded (FFPE) archived pathology samples. From those 68 samples, 19 fresh-frozen tumors were selected for copy number analysis by frequency matching to the larger methylation cohort on age, gender, and stage. Matched peripheral blood was used for SNP-probe normalization. Eleven fresh-frozen non-malignant specimens from the oral cavity, pharynx, and larynx were procured through the National Research Disease Interchange (NRDI).

*DNA Extraction and Array-based Methylation Analysis*. FFPE tumors were sectioned and DNA was isolated, as previously published [20]. DNA was extracted from fresh-frozen tissues and matched peripheral blood samples using the QIAamp DNA mini kit according to the manufacturer's protocol (Qiagen, Valencia, CA). For methylation assessment, sodium bisulfite modification of the DNA was performed using the EZ DNA Methylation Kit (Zymo Research, Orange, CA) with 1 µg of DNA, as described previously [20]. Illumina GoldenGate® methylation bead arrays were used to simultaneously interrogate 1505 CpG loci associated primarily with promoter regions of 803 genes. Arrays were run at the University of California- San Francisco Genomics Core Facility according to the manufacturer's protocol.

*LINE-1 Methylation*. Global DNA methylation was quantified for 11 of the 19 tumor samples with available substrate by pyrosequencing following bisulfite-PCR, with primers and protocols as described in [52]. Four CpG dinucleotides within the human LINE-1 transposon consensus sequence 302–331 (Accession X58075) were analyzed using the PyroMark Q96 MD system. DNA methylation at each locus was calculated by taking the percent of methylated signal divided by the sum of the methylated and unmethylated signals and reported as the mean over all four CpGs. Pyrosequencing reactions were performed in triplicate and bisulfite conversion efficiency was monitored using internal non-CpG cytosine residues.

*SNP Genotyping for Copy Number Status*. Tumors were examined for copy number alterations by hybridizing isolated tumor DNA to the GeneChip® Human Mapping 500 K single-nucleotide polymorphism array (Affymetrix, Santa Clara, CA) following established protocols according to the manufacturer at the Harvard Partners Microarray Core Facility. Probe intensities at each locus were determined in the Affymetrix GeneChip Operating Software and genotypes calls were generated using the Genotyping Analysis Software (Affymetrix). Probe signals were normalized to the matched samples using Copy Number Analysis Tool v4.0.1 [53] (Affymetrix) with the defaults for tuning parameters, Gaussian smoothing, transition decay, and median scaling. Copy number states were inferred by Hidden Markov Model analysis in the same application.

*Statistical Analysis*. BeadStudio software from the array manufacturer Illumina (San Diego, CA) was used for methylation dataset assembly. All array data points are represented by fluorescent signals from both M (methylated) and U (unmethylated) alleles, and methylation level is given by β = (max(*M*, 0))/(|*U*|+|*M*|+100), the average methylation (β) value is derived from the ∼30 replicate methylation measurements and a Cy3/Cy5 methylated/unmethylated ratio. Subsequent analyses were carried out in the R statistical software package (http://www.r-project.org/). For visualization, hierarchical clustering was performed on sample copy number data calls using a Hamming distance metric and calculated by Ward's minimum variance method. Copy number clusters were dichotomized into low and high allelic imbalance (Figure 1A); the difference in absolute mean LINE-1 methylation between the two groups was estimated and confidence intervals were computed. Fisher's exact test for small sample sizes was used to test the degree of abnormal copy number (high/low clustering) for association with the covariates stage (dichotomized as I/II and III/IV), site, and presence of absence of HPV16 viral integration, using a Monte Carlo simulation for site. A Wilcoxon rank-sum test was used to test age as a continuous variable versus degree of copy number variation. For visualization of methylation data, tumors were ordered first by methylation classifications developed in [20] by a recursively partitioned mixture model (RPMM), as described in Houseman *et al.* [31]. Finally, the terminal nodes were obtained by hierarchically clustering methylation β values using Ward's method with a Euclidean distance metric.

Although the 19 tumor samples were the primary focus of this investigation, we used the six classes obtained from the RPMM-clustering on 68 tumors, described in [20], because we anticipated better precision in capturing the true biological inferences with the larger sample size. The relationship between DNA copy number and methylation class membership was tested using the mean value of |CNS-2|, where CNS is the copy number state of each of 500,446 loci. A permutation test with 10 k iterations using the Kruskal-Wallis test statistic was performed. Locus-specific relationships between copy number and methylation were examined graphically in a chromosome-specific manner. Analyses were restricted to autosomal chromosomes. We investigated the local relationship between methylation and CNA at 1413 loci by calculating the Pearson product-moment correlation coefficients. Note that the discrete nature of the Hidden Markov Model motivates the use of the Pearson, rather than Spearman, coefficient. GoldenGate CpG loci were matched to Affymetrix SNPs in the manner described in [32]. In short, each locus was matched to CNS data by selecting the locus having closest HG18/NCBI36 coordinate (typically within 1 kb). *P*-values were calculated via permutation test (5 k permutations). To correct for multiple comparisons, *q*-values were computed by the *qvalue* package in R. To assess correlation at the gene (rather than locus) level, CpGs were matched to genes by chromosomal position, and assigned promoter status if they were upstream of the TSS. Methylation at promoter CpGs were averaged together by gene. Similarly, copy number calls were averaged together for all SNPs associated with a gene. To investigate the molecular processes individually by locus, two-sided, two-sample *t*-tests assuming unequal variance were used to compare methylation between the 19 tumors and 11 non-diseased tissues. For copy number, a one-sample *t*-test was used, with the normal copy number call assumed to be 2. Mean alteration differences were considered statistically significant where *q*<0.05.

## Supporting Information

Figure S1 Integrated color image plots for remaining chromosomes.
(0.88 MB PDF)

Table S1 Top 25 differentially altered genes by DNA methylation and copy number alteration.
(0.03 MB DOC)

Table S2 Local analysis of genes reported to undergo hypermethylation and LOH.
(0.03 MB DOC)
