# Supplementary material for: Genetic and Epigenetic Somatic Alterations in Head and Neck Squamous Cell Carcinomas Are Globally Coordinated but Not Locally Targeted
Source: PLoS One. 2010 Mar 11;5(3):e9651. doi: 10.1371/journal.pone.0009651 (PMC2836370; doi:10.1371/journal.pone.0009651)
Supplement: Figure S1 — Integrated color image plots for remaining chromosomes. (0.88 MB PDF) [file pone.0009651.s001.pdf]

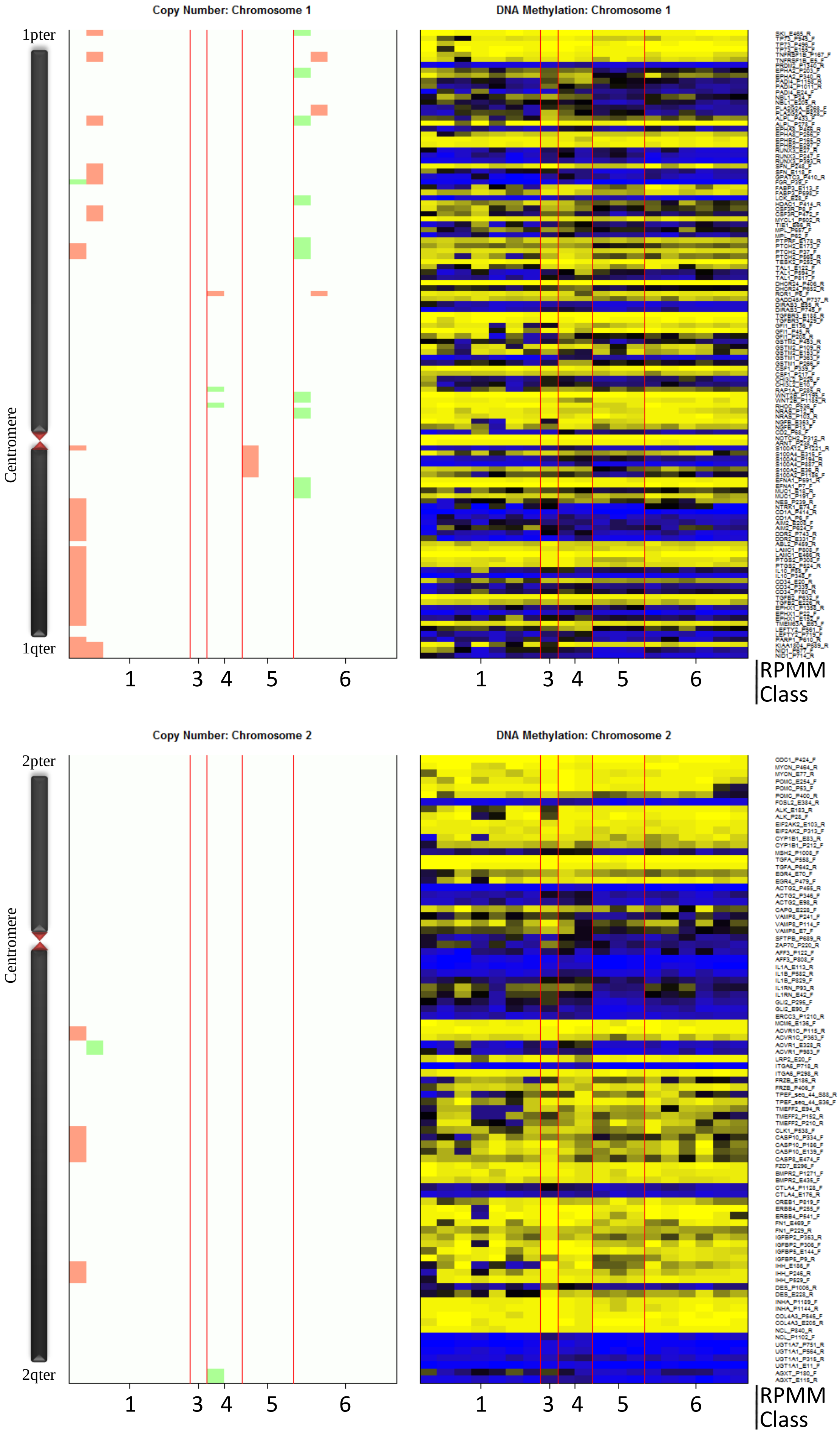

Copy Number: Chromosome 1
DNA Methylation: Chromosome 1
1pter
Centromere
1qter
1
3
4
5
6
RPMM Class
Copy Number: Chromosome 2
DNA Methylation: Chromosome 2
2pter
Centromere
2qter
1
3
4
5
6
RPMM Class

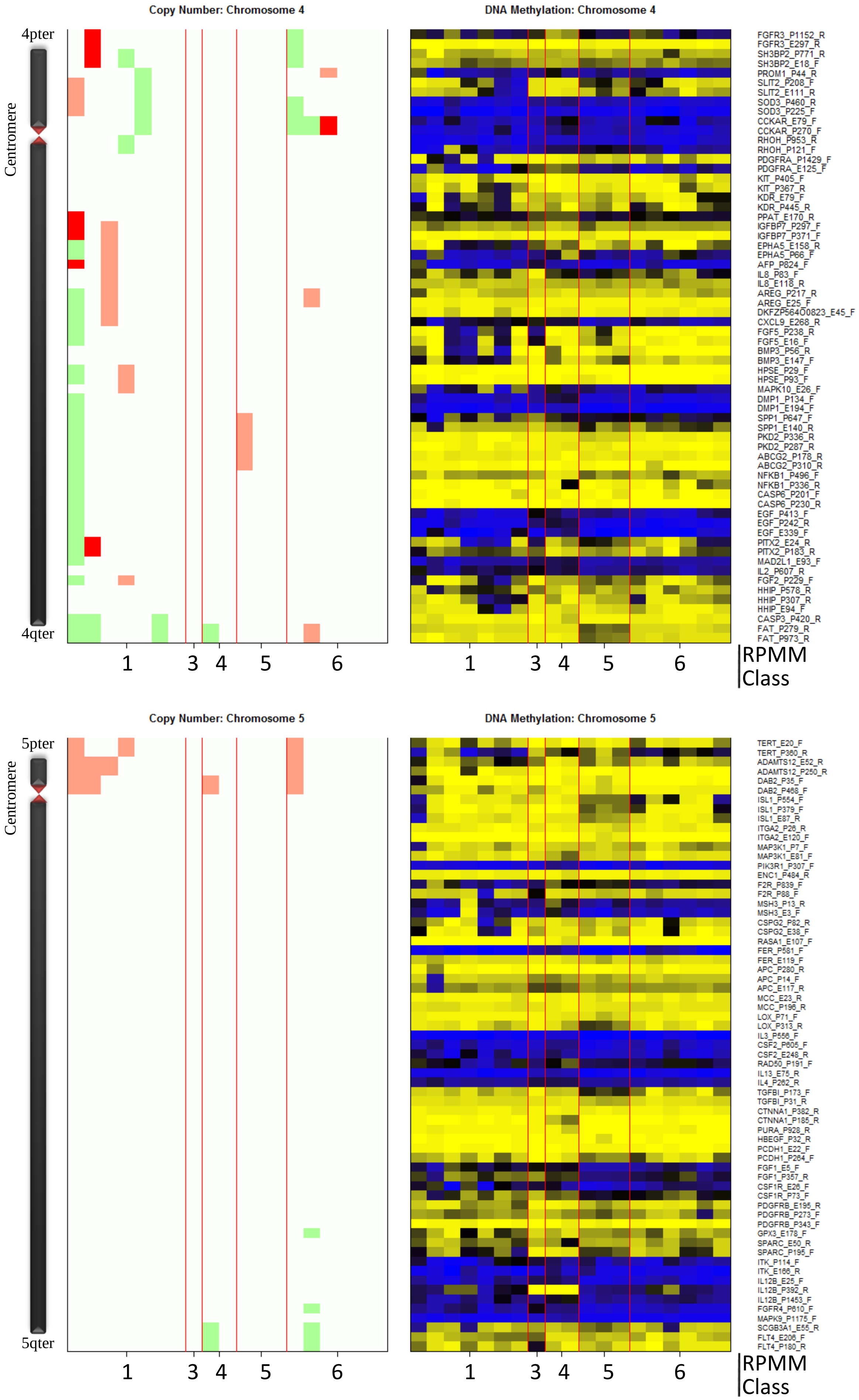
Copy Number: Chromosome 4
DNA Methylation: Chromosome 4
4pter
Centromere
4qter
1
3
4
5
6
RPMM Class
FGFR3_P1152_R
FGFR3_E297_R
SH3BP2_P771_R
SH3BP2_E18_F
PROM1_P44_R
SLIT2_P208_F
SLIT2_E111_R
SOD3_P460_R
SOD3_P225_F
CCKAR_E79_F
CCKAR_P270_F
RHOH_P953_R
RHOH_P121_F
PDGFRA_P1429_F
PDGFRA_E125_F
KIT_P405_F
KIT_P367_R
KDR_E79_F
KDR_P445_R
PPAT_E170_R
IGFBP7_P297_F
IGFBP7_P371_F
EPHA5_E158_R
EPHA5_P66_F
AFP_P824_F
IL8_P83_F
IL8_E118_R
AREG_P217_R
AREG_E25_F
DKFZP564O0823_E45_F
CXCL9_E268_R
FGF5_P238_R
FGF5_E16_F
BMP3_P56_R
BMP3_E147_F
HPSE_P29_F
HPSE_P93_F
MAPK10_E26_F
DMP1_P134_F
DMP1_E194_F
SPP1_P647_F
SPP1_E140_R
PKD2_P336_R
PKD2_P287_R
ABCG2_P178_R
ABCG2_P310_R
NFKB1_P496_F
NFKB1_P336_R
CASP6_P201_F
CASP6_P230_R
EGF_P413_F
EGF_P242_R
EGF_E339_F
PITX2_E24_R
PITX2_P183_R
MAD2L1_E93_F
IL2_P607_R
FGF2_P229_F
HHIP_P578_R
HHIP_P307_R
HHIP_E94_F
CASP3_P420_R
FAT_P279_R
FAT_P973_R
Copy Number: Chromosome 5
DNA Methylation: Chromosome 5
5pter
Centromere
5qter
1
3
4
5
6
RPMM Class
TERT_E20_F
TERT_P360_R
ADAMTS12_E52_R
ADAMTS12_P250_R
DAB2_P35_F
DAB2_P468_F
ISL1_P554_F
ISL1_P379_F
ISL1_E87_R
ITGA2_P26_R
ITGA2_E120_F
MAP3K1_P7_F
MAP3K1_E81_F
PIK3R1_P307_F
ENC1_P484_R
F2R_P839_F
F2R_P88_F
MSH3_P13_R
MSH3_E3_F
CSPG2_P82_R
CSPG2_E38_F
RASA1_E107_F
FER_P581_F
FER_E119_F
APC_P280_R
APC_P14_F
APC_E117_R
MCC_E23_R
MCC_P196_R
LOX_P71_F
LOX_P313_R
IL3_P556_F
CSF2_P605_F
CSF2_E248_R
RAD50_P191_F
IL13_E75_R
IL4_P262_R
TGFBI_P173_F
TGFBI_P31_R
CTNNA1_P382_R
CTNNA1_P185_R
PURA_P928_R
HBEGF_P32_R
PCDH1_E22_F
PCDH1_P264_F
FGF1_E5_F
FGF1_P357_R
CSF1R_E26_F
CSF1R_P73_F
PDGFRB_E195_R
PDGFRB_P273_F
PDGFRB_P343_F
GPX3_E178_F
SPARC_E50_R
SPARC_P195_F
ITK_P114_F
ITK_E166_R
IL12B_E25_F
IL12B_P392_R
IL12B_P1453_F
FGFR4_P610_F
MAPK9_P1175_F
SCGB3A1_E55_R
FLT4_E206_F
FLT4_P180_R

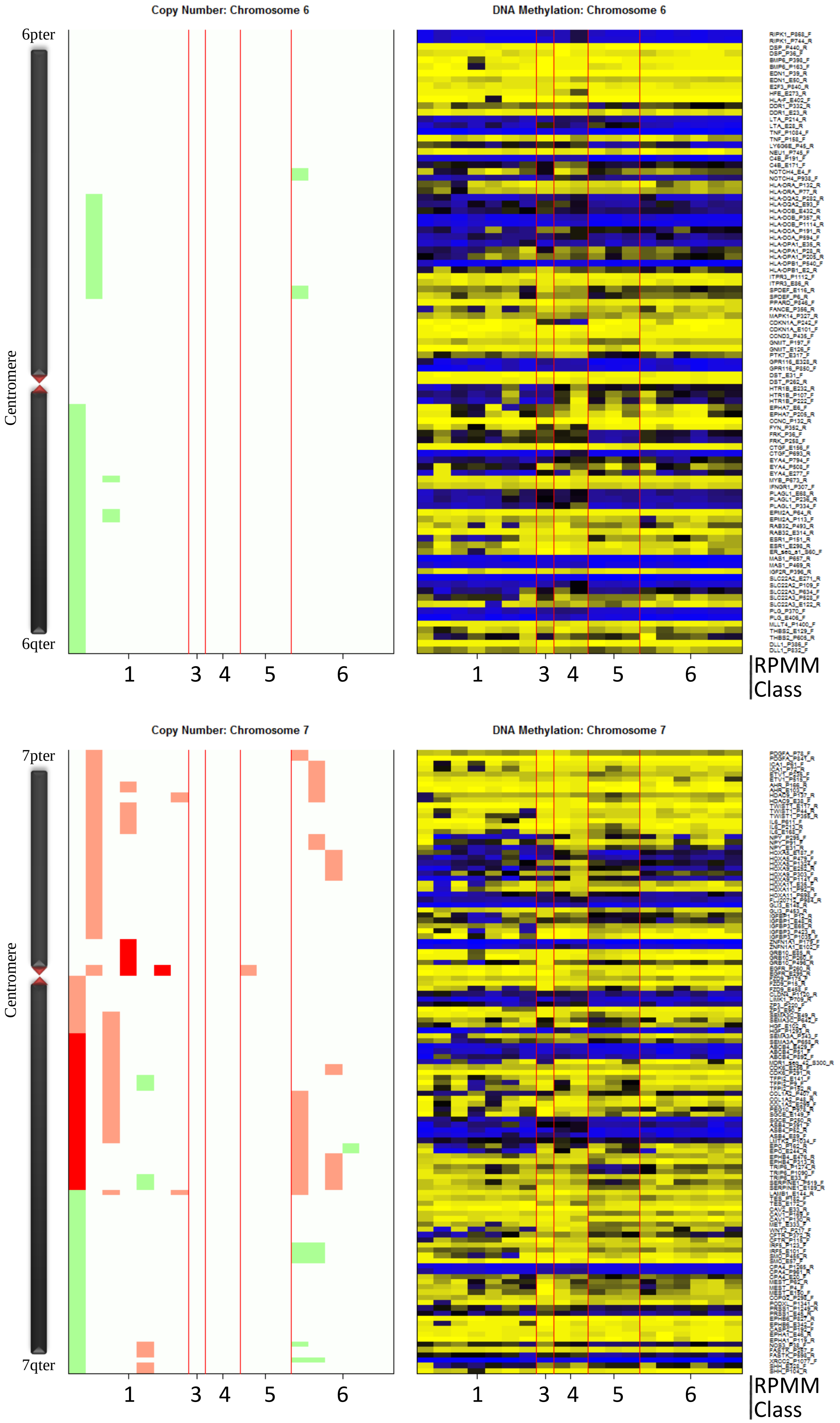

Copy Number: Chromosome 6
DNA Methylation: Chromosome 6
6pter
Centromere
6qter
1
3
4
5
6
RPMM Class
Copy Number: Chromosome 7
DNA Methylation: Chromosome 7
7pter
Centromere
7qter
1
3
4
5
6
RPMM Class

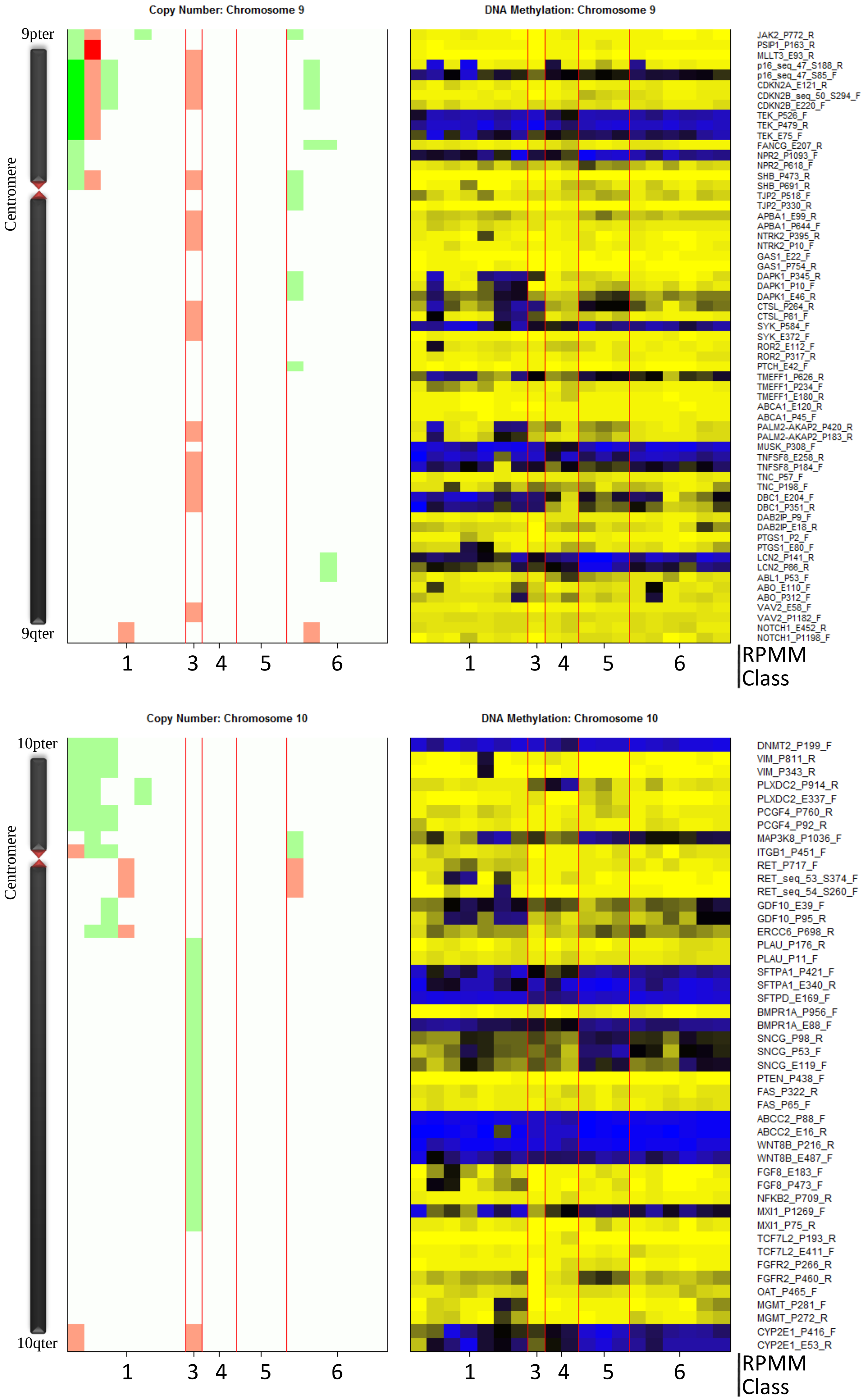
Copy Number: Chromosome 9
DNA Methylation: Chromosome 9
9pter
Centromere
9qter
1
3
4
5
6
RPMM Class
JAK2_P772_R
PSIP1_P163_R
MLLT3_E93_R
p16_seq_47_S188_R
p16_seq_47_S85_F
CDKN2A_E121_R
CDKN2B_seq_50_S294_F
CDKN2B_E220_F
TEK_P526_F
TEK_P479_R
TEK_E75_F
FANCG_E207_R
NPR2_P1093_F
NPR2_P618_F
SHB_P473_R
SHB_P691_R
TJP2_P518_F
TJP2_P330_R
APBA1_E99_R
APBA1_P644_F
NTRK2_P395_R
NTRK2_P10_F
GAS1_E22_F
GAS1_P754_R
DAPK1_P345_R
DAPK1_P10_F
DAPK1_E46_R
CTSL_P264_R
CTSL_P81_F
SYK_P584_F
SYK_E372_F
ROR2_E112_F
ROR2_P317_R
PTCH_E42_F
TMEFF1_P626_R
TMEFF1_P234_F
TMEFF1_E180_R
ABCA1_E120_R
ABCA1_P45_F
PALM2-AKAP2_P420_R
PALM2-AKAP2_P183_R
MUSK_P308_F
TNFSF8_E258_R
TNFSF8_P184_F
TNC_P57_F
TNC_P198_F
DBC1_E204_F
DBC1_P351_R
DAB2IP_P9_F
DAB2IP_E18_R
PTGS1_P2_F
PTGS1_E80_F
LCN2_P141_R
LCN2_P86_R
ABL1_P53_F
ABO_E110_F
ABO_P312_F
VAV2_E58_F
VAV2_P1182_F
NOTCH1_E452_R
NOTCH1_P1198_F
Copy Number: Chromosome 10
DNA Methylation: Chromosome 10
10pter
Centromere
10qter
1
3
4
5
6
RPMM Class
DNMT2_P199_F
VIM_P811_R
VIM_P343_R
PLXDC2_P914_R
PLXDC2_E337_F
PCGF4_P760_R
PCGF4_P92_R
MAP3K8_P1036_F
ITGB1_P451_F
RET_P717_F
RET_seq_53_S374_F
RET_seq_54_S260_F
GDF10_E39_F
GDF10_P95_R
ERCC6_P698_R
PLAU_P176_R
PLAU_P11_F
SFTPA1_P421_F
SFTPA1_E340_R
SFTPD_E169_F
BMPR1A_P956_F
BMPR1A_E88_F
SNCG_P98_R
SNCG_P53_F
SNCG_E119_F
PTEN_P438_F
FAS_P322_R
FAS_P65_F
ABCC2_P88_F
ABCC2_E16_R
WNT8B_P216_R
WNT8B_E487_F
FGF8_E183_F
FGF8_P473_F
NFKB2_P709_R
MXI1_P1269_F
MXI1_P75_R
TCF7L2_P193_R
TCF7L2_E411_F
FGFR2_P266_R
FGFR2_P460_R
OAT_P465_F
MGMT_P281_F
MGMT_P272_R
CYP2E1_P416_F
CYP2E1_E53_R

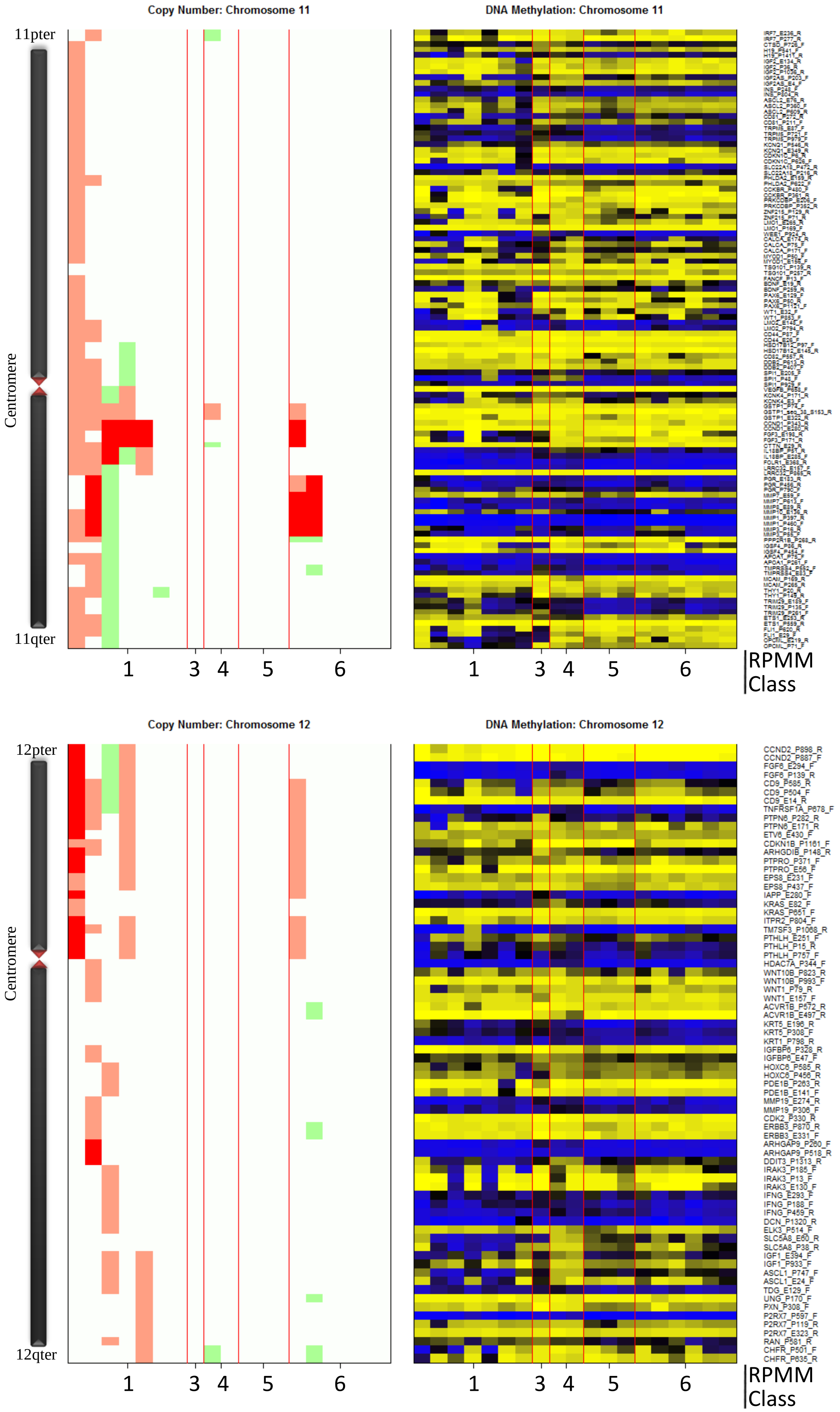

Copy Number: Chromosome 11
DNA Methylation: Chromosome 11
11pter
Centromere
11qter
1
3
4
5
6
1
3
4
5
6
RPMM
Class
Copy Number: Chromosome 12
DNA Methylation: Chromosome 12
12pter
Centromere
12qter
1
3
4
5
6
1
3
4
5
6
RPMM
Class
CCND2_P898_R
CCND2_P887_F
FGF6_E294_F
FGF6_P139_R
CD9_P585_R
CD9_P504_F
CD9_E14_R
TNFRSF1A_P678_F
PTPN6_P282_R
PTPN6_E171_R
ETV6_E430_F
CDKN1B_P1161_F
ARHGDIB_P148_R
PTPRO_P371_F
PTPRO_E56_F
EPS8_E231_F
EPS8_P437_F
IAPP_E280_F
KRAS_E82_F
KRAS_P651_F
ITPR2_P804_F
TM7SF3_P1068_R
PTHLH_E251_F
PTHLH_P15_R
PTHLH_P757_F
HDAC7A_P344_F
WNT10B_P823_R
WNT10B_P993_F
WNT1_P79_R
WNT1_E157_F
ACVR1B_P572_R
ACVR1B_E497_R
KRT5_E196_R
KRT5_P308_F
KRT1_P798_R
IGFBP6_P328_R
IGFBP6_E47_F
HOXC6_P585_R
HOXC6_P456_R
PDE1B_P263_R
PDE1B_E141_F
MMP19_E274_R
MMP19_P306_F
CDK2_P330_R
ERBB3_P870_R
ERBB3_E331_F
ARHGAP9_P260_F
ARHGAP9_P518_R
DDIT3_P1313_R
IRAK3_P185_F
IRAK3_P13_F
IRAK3_E130_F
IFNG_E293_F
IFNG_P188_F
IFNG_P459_R
DCN_P1320_R
ELK3_P514_F
SLC5A8_E60_R
SLC5A8_P38_R
IGF1_E394_F
IGF1_P933_F
ASCL1_P747_F
ASCL1_E24_F
TDG_E129_F
UNG_P170_F
PXN_P308_F
P2RX7_P597_F
P2RX7_P119_R
P2RX7_E323_R
RAN_P581_R
CHFR_P501_F
CHFR_P635_R

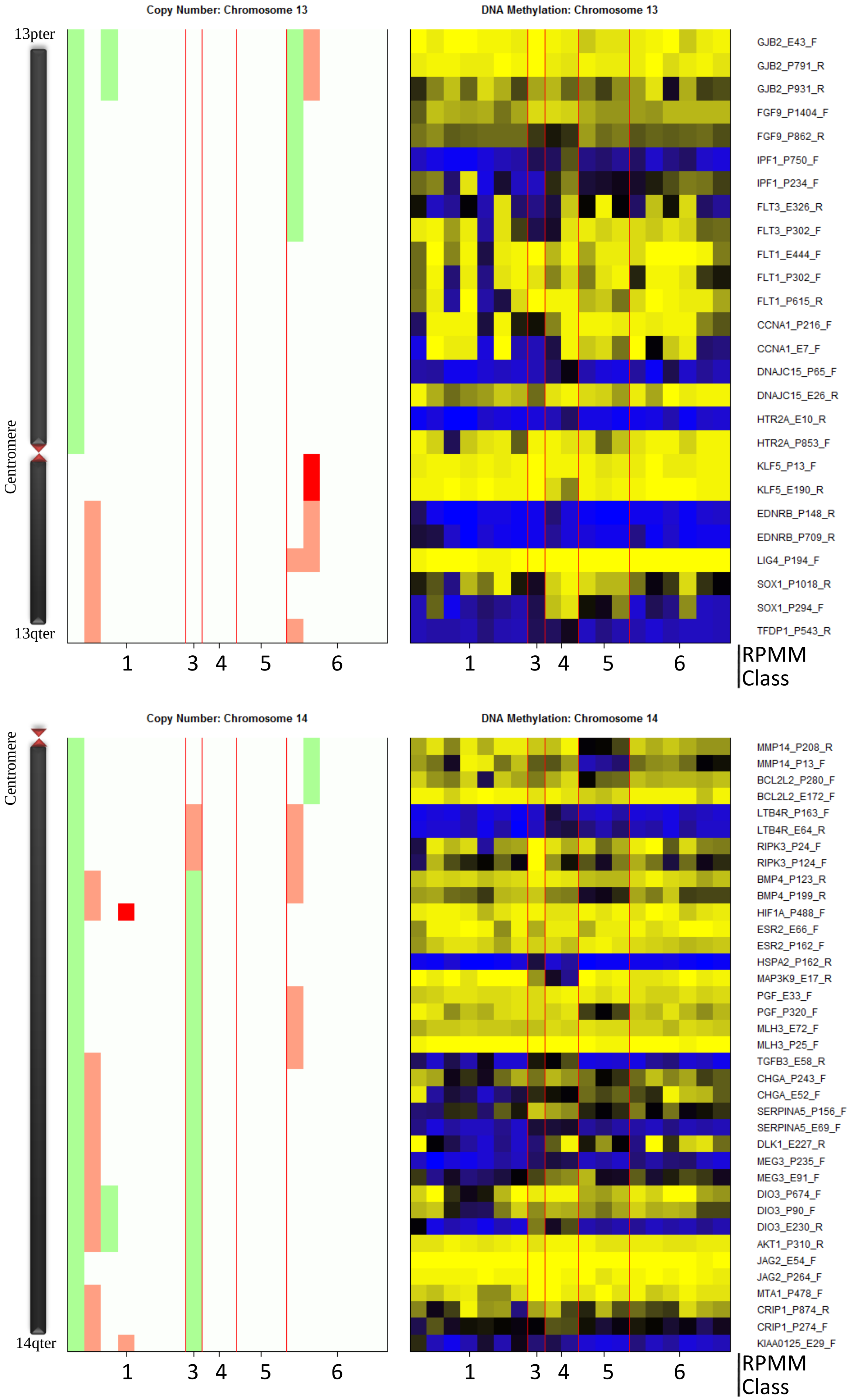
Copy Number: Chromosome 13
DNA Methylation: Chromosome 13
13pter
Centromere
13qter
GJB2_E43_F
GJB2_P791_R
GJB2_P931_R
FGF9_P1404_F
FGF9_P862_R
IPF1_P750_F
IPF1_P234_F
FLT3_E326_R
FLT3_P302_F
FLT1_E444_F
FLT1_P302_F
FLT1_P615_R
CCNA1_P216_F
CCNA1_E7_F
DNAJC15_P65_F
DNAJC15_E26_R
HTR2A_E10_R
HTR2A_P853_F
KLF5_P13_F
KLF5_E190_R
EDNRB_P148_R
EDNRB_P709_R
LIG4_P194_F
SOX1_P1018_R
SOX1_P294_F
TFDP1_P543_R
1 3 4 5 6
1 3 4 5 6
RPMM Class
Copy Number: Chromosome 14
DNA Methylation: Chromosome 14
Centromere
14qter
MMP14_P208_R
MMP14_P13_F
BCL2L2_P280_F
BCL2L2_E172_F
LTB4R_P163_F
LTB4R_E64_R
RIPK3_P24_F
RIPK3_P124_F
BMP4_P123_R
BMP4_P199_R
HIF1A_P488_F
ESR2_E66_F
ESR2_P162_F
HSPA2_P162_R
MAP3K9_E17_R
PGF_E33_F
PGF_P320_F
MLH3_E72_F
MLH3_P25_F
TGFB3_E58_R
CHGA_P243_F
CHGA_E52_F
SERPINA5_P156_F
SERPINA5_E69_F
DLK1_E227_R
MEG3_P235_F
MEG3_E91_F
DIO3_P674_F
DIO3_P90_F
DIO3_E230_R
AKT1_P310_R
JAG2_E54_F
JAG2_P264_F
MTA1_P478_F
CRIP1_P874_R
CRIP1_P274_F
KIAA0125_E29_F
1 3 4 5 6
1 3 4 5 6
RPMM Class

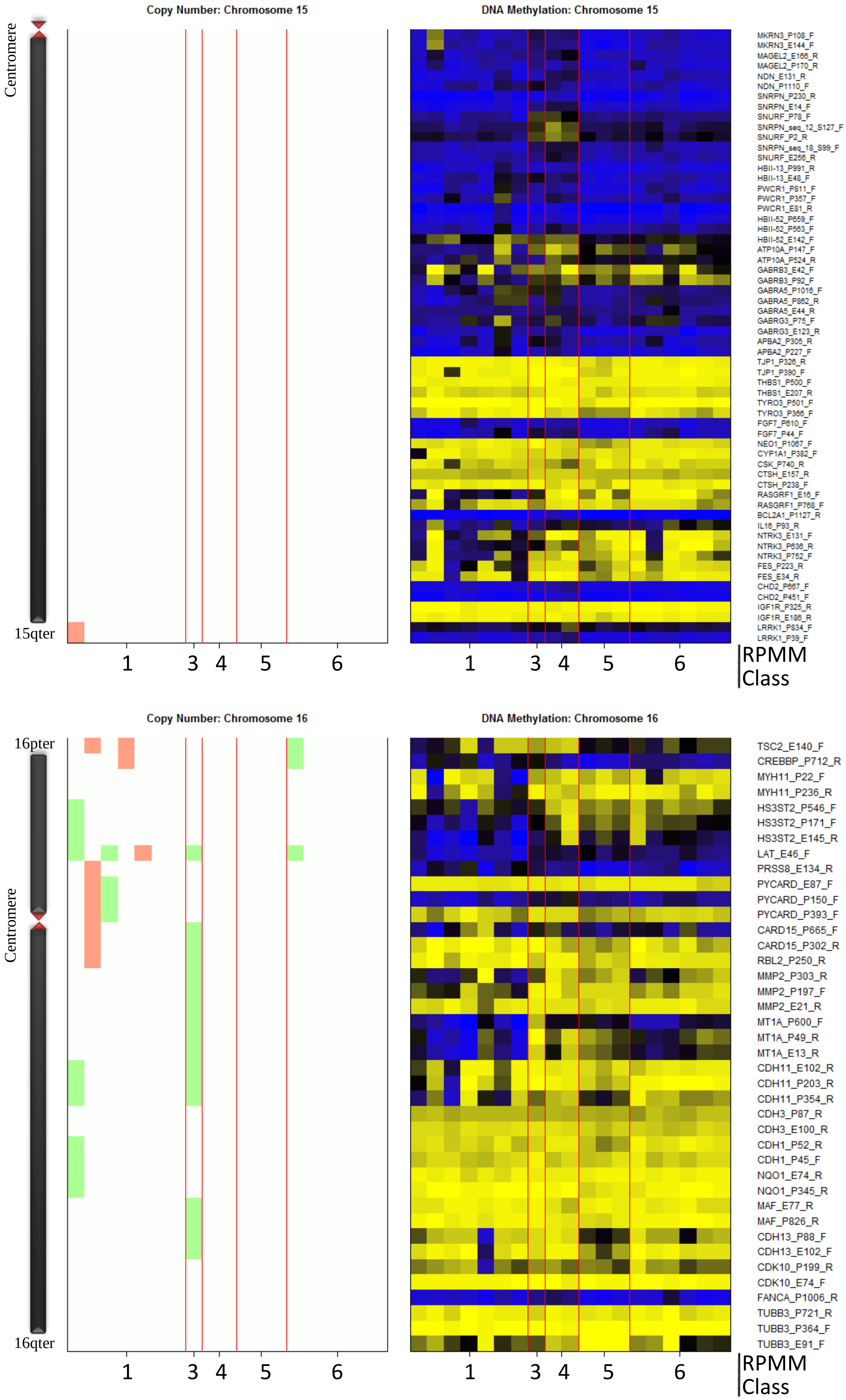
Copy Number: Chromosome 15
DNA Methylation: Chromosome 15
Centromere
15qter
MKRN3_P108_F
MKRN3_E144_F
MAGEL2_E166_R
MAGEL2_P170_R
NDN_E131_R
NDN_P1110_F
SNRPN_P230_R
SNRPN_E14_F
SNURF_P78_F
SNRPN_seq_12_S127_F
SNURF_P2_R
SNRPN_seq_18_S99_F
SNURF_E256_R
HBII-13_P991_R
HBII-13_E48_F
PWCR1_P811_F
PWCR1_P357_F
PWCR1_E81_R
HBII-52_P659_F
HBII-52_P563_F
HBII-52_E142_F
ATP10A_P147_F
ATP10A_P524_R
GABRB3_E42_F
GABRB3_P92_F
GABRA5_P1016_F
GABRA5_P862_R
GABRA5_E44_R
GABRG3_P75_F
GABRG3_E123_R
APBA2_P305_R
APBA2_P227_F
TJP1_P326_R
TJP1_P390_F
THBS1_P500_F
THBS1_E207_R
TYRO3_P501_F
TYRO3_P366_F
FGF7_P610_F
FGF7_P44_F
NEO1_P1067_F
CYP1A1_P382_F
CSK_P740_R
CTSH_E157_R
CTSH_P238_F
RASGRF1_E16_F
RASGRF1_P768_F
BCL2A1_P1127_R
IL16_P93_R
NTRK3_E131_F
NTRK3_P636_R
NTRK3_P752_F
FES_P223_R
FES_E34_R
CHD2_P667_F
CHD2_P451_F
IGF1R_P325_R
IGF1R_E186_R
LRRK1_P834_F
LRRK1_P39_F
1 3 4 5 6
1 3 4 5 6
RPMM Class
Copy Number: Chromosome 16
DNA Methylation: Chromosome 16
16pter
Centromere
16qter
TSC2_E140_F
CREBBP_P712_R
MYH11_P22_F
MYH11_P236_R
HS3ST2_P546_F
HS3ST2_P171_F
HS3ST2_E145_R
LAT_E46_F
PRSS8_E134_R
PYCARD_E87_F
PYCARD_P150_F
PYCARD_P393_F
CARD15_P665_F
CARD15_P302_R
RBL2_P250_R
MMP2_P303_R
MMP2_P197_F
MMP2_E21_R
MT1A_P600_F
MT1A_P49_R
MT1A_E13_R
CDH11_E102_R
CDH11_P203_R
CDH11_P354_R
CDH3_P87_R
CDH3_E100_R
CDH1_P52_R
CDH1_P45_F
NQO1_E74_R
NQO1_P345_R
MAF_E77_R
MAF_P826_R
CDH13_P88_F
CDH13_E102_F
CDK10_P199_R
CDK10_E74_F
FANCA_P1006_R
TUBB3_P721_R
TUBB3_P364_F
TUBB3_E91_F
1 3 4 5 6
1 3 4 5 6
RPMM Class

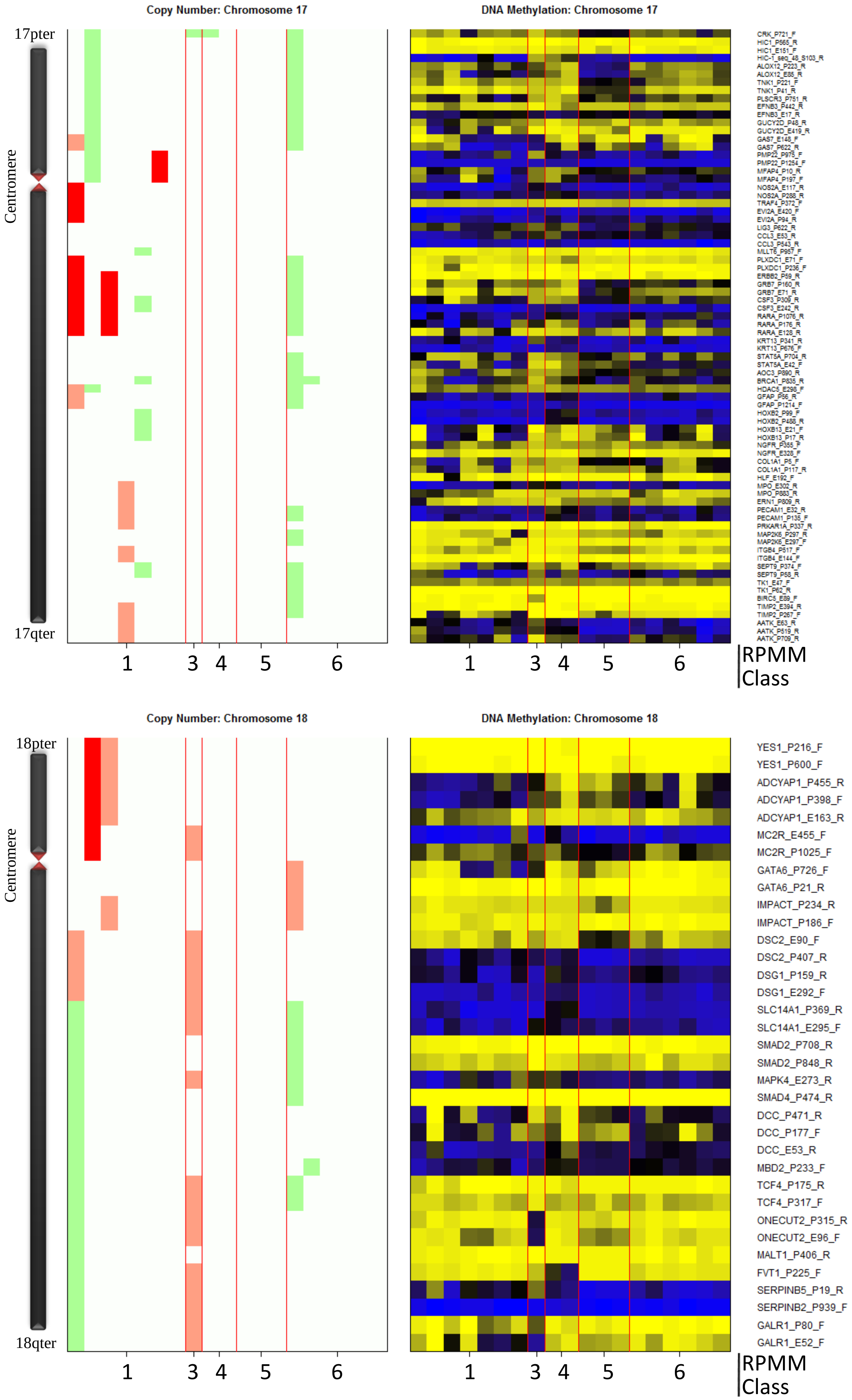

Copy Number: Chromosome 17
DNA Methylation: Chromosome 17
17pter
Centromere
17qter
1
3
4
5
6
CRK_P721_F
HIC1_P565_R
HIC1_E151_F
HIC-1_seq_48_S103_R
ALOX12_P223_R
ALOX12_E85_R
TNK1_P221_F
TNK1_P41_R
PLSCR3_P751_R
EFNB3_P442_R
EFNB3_E17_R
GUCY2D_P48_R
GUCY2D_E419_R
GAS7_E148_F
GAS7_P622_R
PMP22_P975_F
PMP22_P1254_F
MFAP4_P10_R
MFAP4_P197_F
NOS2A_E117_R
NOS2A_P288_R
TRAF4_P372_F
EVI2A_E420_F
EVI2A_P94_R
LIG3_P622_R
CCL3_E53_R
CCL3_P543_R
MLLT6_P957_F
PLXDC1_E71_F
PLXDC1_P236_F
ERBB2_P59_R
GRB7_P160_R
GRB7_E71_R
CSF3_P309_R
CSF3_E242_R
RARA_P1076_R
RARA_P176_R
RARA_E128_R
KRT13_P341_R
KRT13_P676_F
STAT5A_P704_R
STAT5A_E42_F
AOC3_P890_R
BRCA1_P835_R
HDAC5_E298_F
GFAP_P56_R
GFAP_P1214_F
HOXB2_P99_F
HOXB2_P488_R
HOXB13_E21_F
HOXB13_P17_R
NGFR_P355_F
NGFR_E328_F
COL1A1_P5_F
COL1A1_P117_R
HLF_E192_F
MPO_E302_R
MPO_P883_R
ERN1_P809_R
PECAM1_E32_R
PECAM1_P135_F
PRKAR1A_P337_R
MAP2K6_P297_R
MAP2K6_E297_F
ITGB4_P517_F
ITGB4_E144_F
SEPT9_P374_F
SEPT9_P58_R
TK1_E47_F
TK1_P62_R
BIRC5_E89_F
TIMP2_E394_R
TIMP2_P267_F
AATK_E63_R
AATK_P519_R
AATK_P709_R
RPMM Class
Copy Number: Chromosome 18
DNA Methylation: Chromosome 18
18pter
Centromere
18qter
YES1_P216_F
YES1_P600_F
ADCYAP1_P455_R
ADCYAP1_P398_F
ADCYAP1_E163_R
MC2R_E455_F
MC2R_P1025_F
GATA6_P726_F
GATA6_P21_R
IMPACT_P234_R
IMPACT_P186_F
DSC2_E90_F
DSC2_P407_R
DSG1_P159_R
DSG1_E292_F
SLC14A1_P369_R
SLC14A1_E295_F
SMAD2_P708_R
SMAD2_P848_R
MAPK4_E273_R
SMAD4_P474_R
DCC_P471_R
DCC_P177_F
DCC_E53_R
MBD2_P233_F
TCF4_P175_R
TCF4_P317_F
ONECUT2_P315_R
ONECUT2_E96_F
MALT1_P406_R
FVT1_P225_F
SERPINB5_P19_R
SERPINB2_P939_F
GALR1_P80_F
GALR1_E52_F
1
3
4
5
6
RPMM Class

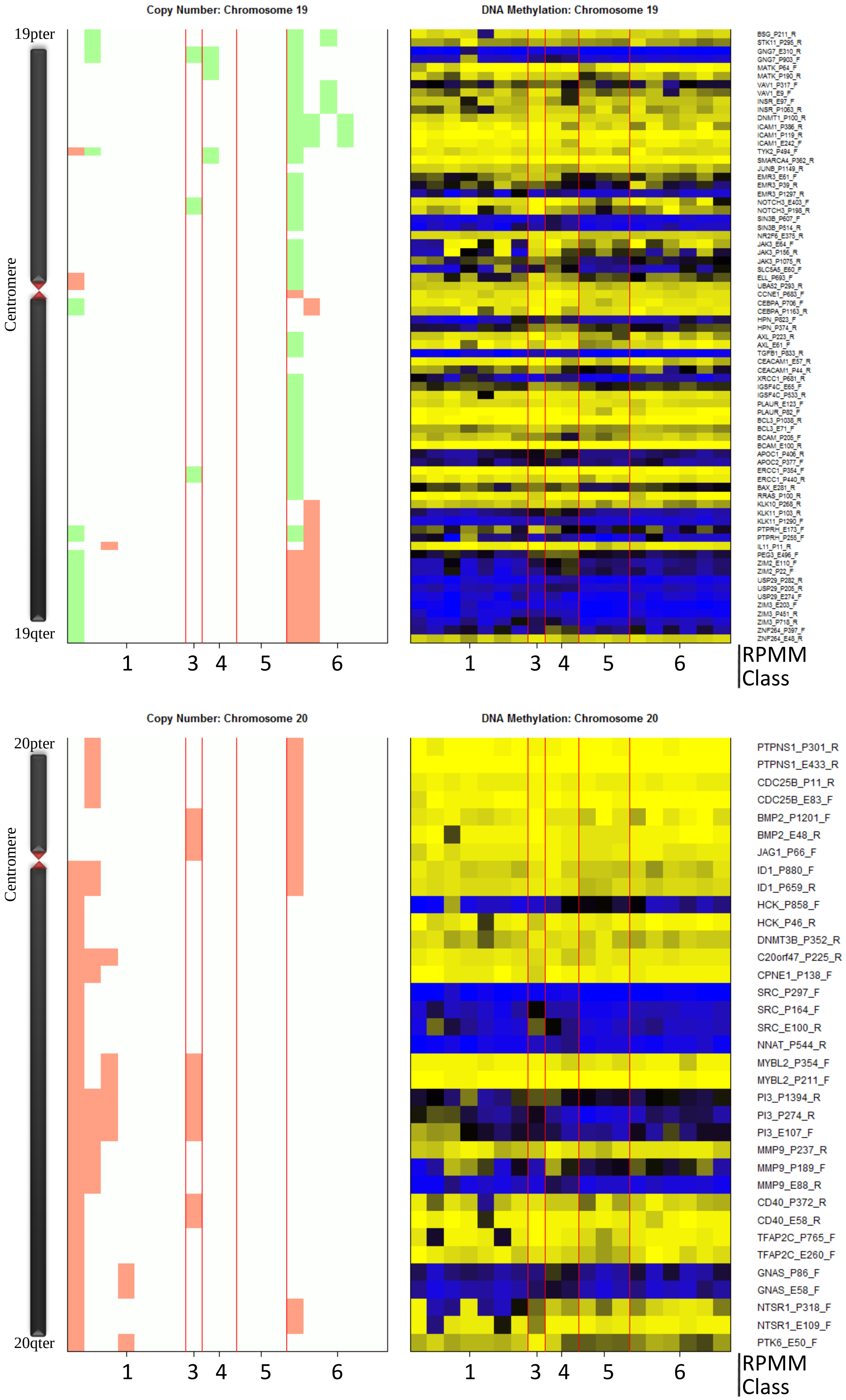
Copy Number: Chromosome 19
DNA Methylation: Chromosome 19
19pter
Centromere
19qter
1 3 4 5 6
1 3 4 5 6
RPMM Class
Copy Number: Chromosome 20
DNA Methylation: Chromosome 20
20pter
Centromere
20qter
PTPNS1_P301_R
PTPNS1_E433_R
CDC25B_P11_R
CDC25B_E83_F
BMP2_P1201_F
BMP2_E48_R
JAG1_P66_F
ID1_P880_F
ID1_P659_R
HCK_P858_F
HCK_P46_R
DNMT3B_P352_R
C20orf47_P225_R
CPNE1_P138_F
SRC_P297_F
SRC_P164_F
SRC_E100_R
NNAT_P544_R
MYBL2_P354_F
MYBL2_P211_F
PI3_P1394_R
PI3_P274_R
PI3_E107_F
MMP9_P237_R
MMP9_P189_F
MMP9_E88_R
CD40_P372_R
CD40_E58_R
TFAP2C_P765_F
TFAP2C_E260_F
GNAS_P86_F
GNAS_E58_F
NTSR1_P318_F
NTSR1_E109_F
PTK6_E50_F
1 3 4 5 6
1 3 4 5 6
RPMM Class

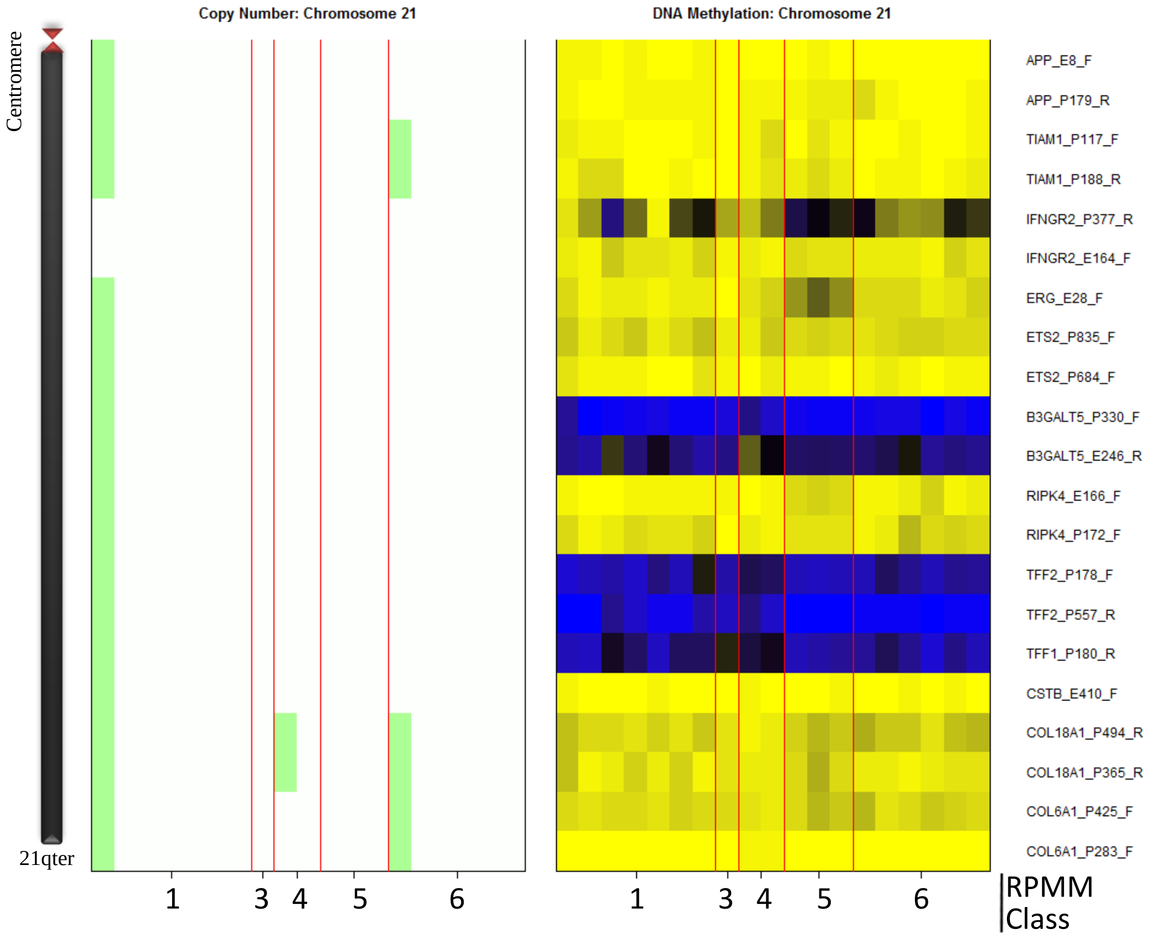

Copy Number: Chromosome 21
DNA Methylation: Chromosome 21
Centromere
21qter
1
3
4
5
6
RPMM Class
APP_E8_F
APP_P179_R
TIAM1_P117_F
TIAM1_P188_R
IFNGR2_P377_R
IFNGR2_E164_F
ERG_E28_F
ETS2_P835_F
ETS2_P684_F
B3GALT5_P330_F
B3GALT5_E246_R
RIPK4_E166_F
RIPK4_P172_F
TFF2_P178_F
TFF2_P557_R
TFF1_P180_R
CSTB_E410_F
COL18A1_P494_R
COL18A1_P365_R
COL6A1_P425_F
COL6A1_P283_F

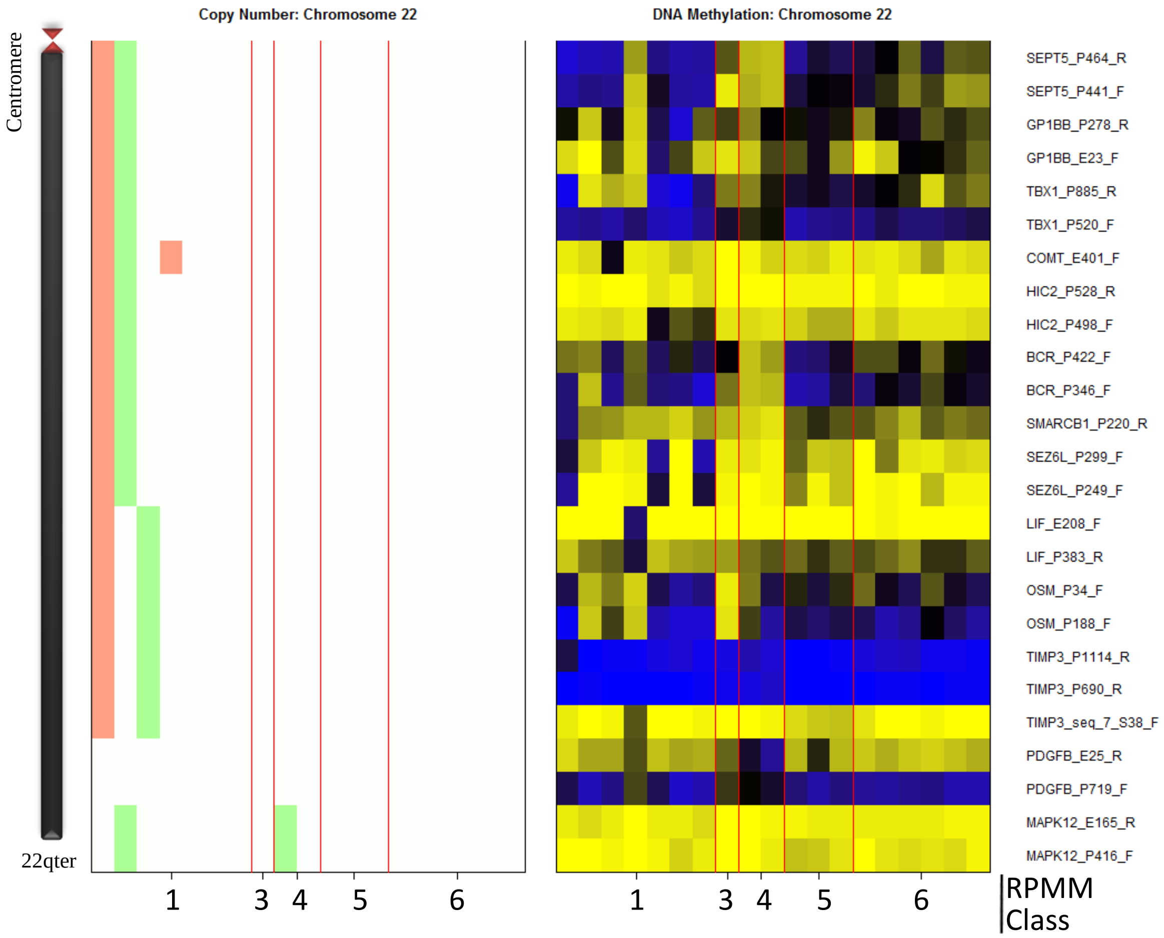

Copy Number: Chromosome 22
DNA Methylation: Chromosome 22
Centromere
22qter
1
3
4
5
6
RPMM Class
SEPT5_P464_R
SEPT5_P441_F
GP1BB_P278_R
GP1BB_E23_F
TBX1_P885_R
TBX1_P520_F
COMT_E401_F
HIC2_P528_R
HIC2_P498_F
BCR_P422_F
BCR_P346_F
SMARCB1_P220_R
SEZ6L_P299_F
SEZ6L_P249_F
LIF_E208_F
LIF_P383_R
OSM_P34_F
OSM_P188_F
TIMP3_P1114_R
TIMP3_P690_R
TIMP3_seq_7_S38_F
PDGFB_E25_R
PDGFB_P719_F
MAPK12_E165_R
MAPK12_P416_F
